# The Royal British Nurses' Association

**Published:** 1917-11-10

**Authors:** 


### THE ROYAL BRITISH NURSES' ASSOCIATION.

### The Story of Recent Happenings.

THE *Nurses' Journal*, the official organ of the Royal British Nurses' Association, in its November issue* contains an editorial article, approved by the Executive Committee, dealing with the Supplemental Charter; a copy of the Draft Supplemental Charter (Extension of Powers and Purposes of the Corporation) (*a*) as originally drafted, and (*b*) as amended by the Lords of the Privy Council; a report of the meeting of the General Council on July 5 last; a correspondence between the solicitors of the Royal British Nurses' Association and Sir Almeric Fitzroy, the Clerk to the Privy Council; and a report of the special meeting of the General Council held on September 27 last, which includes a memorandum embodying a statement of some Nurse-Members of the Council on certain points in connection with the College of Nursing and themselves, which they declared were not very clear to them. This memorandum, we may point out in passing, contains two material misstatements. One is that, under the unamended Charter as it stands, the Royal British Nurses' Association can keep a Register, whereas in fact the Charter confers no such power. Secondly, that the College authorities have made decisions and held conferences affecting the future of the conjoint body without consulting or even informing the Executive Committee of the Royal British Nurses' Association. This, we are assured, is not the fact, nor has any step of the kind been taken by the College. The compilers of the memorandum, therefore, were evidently under a serious misapprehension as to the position taken up by the representatives of the College in regard to the Register of Nurses formed by the College, the effects of the amalgamation of the Royal British Nurses' Association with this body, and the addition of the names of its members to this Register. This memorandum contains other evidence of serious misapprehension and misunderstanding of the actual position, which are apparent on the face of it.

We publish in another column the full report of the meeting of the Council on July 5, that members of the nursing profession may have an opportunity of appreciating these misapprehensions by reading the report of the discussion and of the remarks made by Sir James Crichton Browne, F.R.S., the chairman of that meeting, whose knowledge of public business and affairs, and the bearings of Charters and other public documents, is exceptionally great and unusually complete. For the rest it will be seen that at the meeting of the Council on July 5 there were apparently only thirteen present, of whom eight voted for the postponement for practically three months of the consideration of the amendments made in the Draft Supplemental Charter by the Privy Council, and five voted against it. At the meeting of the Council on September 27 there were apparently eighteen present, in addition to the honorary officers; two members, with the honorary officers, did not vote, whilst the remaining sixteen Nurse-Members present voted for the resolution already published in these columns (see THE HOSPITAL, October 6, 1917, page 17), which declared it would not be to the interests of the Corporation to accept the alterations suggested—*i.e.* by the Privy Council.

On the face of it, it must be apparent to those intimately acquainted with Royal Charters and the legal aspects which affect them and their amendment, that the Nurse-Members could not, as is natural, have realised the true bearings of the matters before the Council, as Sir James Crichton Browne, their chairman, with great patience and knowledgeable apprehension, carefully explained and insisted on. Had they or their advisers understood the matter in all its aspects, and the true bearing of the amendments made by the Privy Council, as subsequently modified, the Nurse-Members, in the best interests of the members of the R.B.N.A. and its future, must have hesitated and, as we believe, have declined to adopt the decision, embodied in the resolution actually passed by the votes of the nurses.

We lament that the good intentions on both sides with which the scheme to amalgamate was entered upon should have failed to materialise. The views of the College of Nursing have not yet been published, and our readers will note that there is still time, all too brief as it is, for reconsideration on the part of the sixteen nurses who have delayed the work of the College and placed the Royal British Nurses' Association and its Royal Charter in what amounts in fact to a *cul de sac*.

The sympathy of the whole nursing world has gone out to H.R.H. Princess Christian in the loss of her husband, and we hope, as all her well-wishers will hope, that the Princess may be well enough to give the most earnest consideration to the present very serious position which her Royal Charter and Association occupy to-day. H.R.H. has been the backbone and strength of this Chartered Society, and it would be lamentable. indeed, if all her interest and self-denying efforts should be imperilled by misapprehensions, and a want of technical knowledge and confidence which ought not to have been possible in the conduct of a business of such importance to all concerned. Meanwhile we hope our readers will really study carefully the report we republish of the meeting last July, which will enable the nursing profession as a whole to realise the position which has brought about the present *impasse* in the affairs of the Royal British Nurses' Association.

* A limited number of copies of this publication can be obtained from the offices of the Royal British Nurses' Association, 10 Orchard Street, London, W., price 2d. each, or by post 2½d.

### The Royal British Nurses' Association.

### COUNCIL MEETING, JULY 5th, 1917.

### Report of Proceedings in so far as they relate to the Supplemental Charter (from the *Nurses' Journal*, November 1917).

Mr. Paterson having read the letter from the Privy Council dated June 13 and drawn attention to the suggested alterations on the Draft Supplemental Charter, as submitted to the meeting, the Chairman, Sir James Crichton Browne, said: I think the points which demand consideration are those to which Mr. Paterson has called your attention. Clause D ran as follows in your Supplemental Charter: "The making and maintaining of an Official Register of persons qualified to act as Nurses." The Privy Council previously struck out the words "the Official Register" and substituted "a Roll." I have no doubt the word "Roll" was suggested to the Council by the Roll of Midwives, and I think, owing to the action of your Honorary Secretary and Mr. Stanley, the Privy Council have given way upon that point, and have agreed that the clause shall run as follows: "The making and maintaining of a Register of persons qualified to act as Nurses." It seems to me that the retention of the word "Register" instead of the word "Roll" and without the prefix "Official" in Section 2, Clause D, meets the legitimate requirements of the Corporation. The Register which it would be the purpose of the Corporation to make and maintain would be official so far as the Association is concerned, and if we did not obtain the Supplemental Charter we should not be entitled to use the word "Register," but must fall back on the word "list," which has no official significance. I think it is a great point gained that the Privy Council gave way on this.

As regards the words in the first two lines of Clause E of Section 2 which read: "to promote legislation to provide for the State recognition of and protection of the Official Register," the Privy Council suggest that the word "an" be substituted for "the" before "Official Register." They propose that a Register of one kind or another shall be kept. Now I understand that it has been suggested that this Supplemental Charter is an attempt to forestall Parliament. As regards that I would observe that the first line of Clause E, Section 2, "to promote legislation," etc., cannot be construed into an attempt to forestall the action of Parliament, and should legislation for the Official or Statutory Registration of Nurses be undertaken, it will be for Parliament to define what that Register shall be and the qualifications of those whose names shall be inscribed upon it, but it will be within the competency of any association or any individual to promote the recognition and protection of any existing Register, if, however, it be that the word "Official" is objected to, as liable to be understood to imply some existing claim to statutory recognition, then the substitution for the words "the Official Register" of the words "the Register of the Corporation" should, I think, meet the views of the Privy Council. Of course, if the Privy Council agree that we are to have a Register, they admit that it becomes a Register not merely of the British Nurses' Association but of the combined body, and I think that is a strong position. They left the clause in a much more favourable form than I expected. If they accept the words: "The State recognition and protection of the Register of the Corporation," this will give you a status against any other Register; it will practically grant you "protection for the Register of the Corporation."

Dr. Stewart: Many nurses, as I understand, were misled by the circular of the College, and I feel that it would not do to consent to these alterations while they still have this idea. They ought to have opportunity for expressing their opinions.

Sir James Crichton Browne: Unfortunately that appears to have been so, but if you carry out your amalgamation you will have 4,000 members of the British Nurses' Association and 5,000 of the College of Nursing —9,000 nurses. It is certain that with that number on the Register it will be accepted as an accomplished fact by the State. It will become the basis of the State Register.

Mrs. Latter: This does not seem secure, and we ought to have some sort of assurance. I think that we must support the College in regard to this matter. If we allow a loophole of any kind we must be certain of our position in the matter.

Mr. Berkeley: This does not seem essential, as they cannot take any action until the Charter is passed.

Mrs. Latter: I think we have some responsibility in regard to them.

Mr. Berkeley: The College did not promise that their nurses would be on the State Register. They could not promise it.

A Member: They only promised to draft a Bill. Any sensible woman reading that would understand that till the Bill was passed they could not come into that.

Sir James Crichton Browne: I think it is certain that the Register would be accepted.

Mrs. Latter: The first clause on the College paper runs to the effect that theirs shall be the first Register under the Act.

Sir James Crichton Browne: I take it that the Privy Council will not include the words "the Official Register" in Clause E.

Mrs. Latter: Then, I think, that we are not taking powers to fulfil all pledges.

Sir James Crichton Browne: The Privy Council evidently think that the word "official" may be understood to mean statutory. The word "official" is used a great deal in Government offices—a letter is called "official." It is a mere indication that it has formal recognition. I think it is a great pity to risk the amalgamation breaking down for a mere quibble. I am of the opinion that the Privy Council will not give way on this small point.

Mrs. Latter: The whole thing is extremely involved, and I think that it would be well not to rush it through. How would it be to hang it up for a little while and take time to consider it? I think rushed legislation is always rash legislation.

A Member: I think that you cannot blame us if we have taken the paragraph in the prospectus literally as other nurses have done. Could the Local Government set up an official Register?

Mr. Paterson: I do not think so without the sanction of Parliament. There is no getting away from the fact that this Supplemental Charter was meant to ensure, as far as may be possible, that the Conjoint Register should

ROYAL BRITISH NURSES' ASSOCIATION—(*cont.*)

be the State Register. If, as is now stated, it was so obvious that Parliament would not be "forestalled," I cannot understand why the Supplemental Charter was drafted as it is, and why matters were allowed to proceed so far. However, I do not agree that we are jeopardising anything by delay. We can amalgamate to-morrow. Our existing Charter gives us the power to do so. We are simply taking time to consider, and we must keep faith with our nurses. If Mrs. Latter is going to propose that the matter be deferred, if the opinion of the majority is that the matter should be deferred, well, we must discuss it another time.

Sir James Crichton Browne: There is no need for this, it is a quibble.

Mr. Paterson: I regret that I cannot agree with you, Sir. I consider that a principle is involved.

Sir James Crichton Browne: What is the principle?

Mr. Paterson: The principle is that the nurses have understood that they are going automatically to be put on the State Register, and that they understood that we were taking powers to promote our Register as the official one.

Sir James Crichton Browne: But you are obliged to go through with the amalgamation. The Privy Council expects an answer.

Mrs. Latter: The Privy Council are doubtless like other Government Departments, and, therefore, can be in no hurry.

Sir James Crichton Browne: It is not a substantial ground. It is only formal whether it stands as the Register of the Corporation or the Official Register. I think it is very satisfactory that the Privy Council gave up the word "Roll" and adopted the word "Register."

Mrs. Latter: Need we hurry to a decision? I strongly deprecate any premature action in the matter, though I think we must adhere to the agreed terms.

Sir James Crichton Browne: Register and Official Register are identical. I think it is very important that this thing should proceed. The Long Vacation is coming on, and I think we ought not to risk a delay.

Mrs. Latter: I think the matter calls for time for consideration.

Mr. Berkeley: It seems important that we should try to conclude this as soon as we can for the sake of the Royal British Nurses' Association.

A Member: But more for the sake of the College of Nursing.

Sir James Crichton Browne: The proposed Register of the Corporation would be official in as far as the Association is concerned. The Society for the State Registration of Nurses objects to the word "official."

Mr. Paterson: Pardon me, Sir James, the objection was raised by the Local Government Board. I have a letter from our solicitors to this effect.

Sir James Crichton Browne: Will you read us this letter?

(The solicitor's letter was read to the meeting by Mr. Paterson.)

Mr. Paterson: I think it a very good suggestion that we should have more time to consider. Many of the nurses feel that they do not understand the points clearly. As regards the Supplemental Charter, I and they think that it was meant to "forestall" Parliament.

Sir James Crichton Browne: Do you really think that you can "forestall" Parliament?

Mr. Paterson: That is not the point. The point is that the College have done so by giving their pledge that the nurses will be on the State Register.

Sir James Crichton Browne: We are not responsible for this.

A Member: We shall be when we take in the College.

Sir James Crichton Browne: I should think that no promise could be given by any public body—(interruption, "But they did!")—but there is every probability that this will be fulfilled. Parliament could not ignore a Register of 9,000 nurses.

Mrs. Latter: I consider that the matter should be postponed to a later date, and that if this were done it would facilitate the harmony of this meeting.

At the request of Sir James Crichton Browne, Mr. Paterson read the letter which had been drafted as a reply to the Privy Council.

Mr. Berkeley: I should like to know whether the members really think that we could force Parliament to accept our Register.

A Member: We do not think so any longer, but we were under that impression. It is not, surely, surprising that if the Privy Council took the Supplemental Charter as an attempt to "forestall" Parliament we nurses should do the same.

Sir James Crichton Browne: The proper way to bring it to an issue is that I move from the Chair that the amendments suggested by the Privy Council be accepted, and then Mrs. Latter can move an amendment that the question be postponed for consideration later.

Sir James Crichton Browne then proposed that the amendments suggested by the Privy Council be accepted.

Mrs. Latter proposed as an amendment that the whole matter be postponed for consideration at a future meeting. This was seconded and put to the meeting. Eight voted for the amendment and five against it.

The amendment was therefore carried and was put as a substantive motion and carried with two dissentients.

Sir James Crichton Browne: I presume that another meeting will be called. I confess that I am disappointed, very disappointed.

A Member: When I first saw the College paper I thought it would make all the difference to the nursing profession, as Registration with some body like the College was in my mind. I am an old member of the Royal British Nurses' Association, and when I came up in January I certainly understood that the aim was an official Register, and so I wrote to quite fifty of my old nurses and put the whole thing before them. I also wrote to many on foreign service. They said that they did not see the need for joining the College; many of them were coming to the end of their careers, and guineas were not too plentiful with some of them. However, many of them wrote thanking me, and I have a letter from the College saying "you are helping us splendidly." Altogether I got a lot of new members for the College. But as an old member of the Royal British Nurses' Association, I feel that we are in need of some safeguard with regard to our position, and I particularly wish to emphasise this.

Sir James Crichton Browne: I think it is most unfortunate that the impression should have been created, and I cannot conceive the impression being made. I think that Registration was never more desired than at the present time. Thousands of V.A.D.s will be coming home and posing as nurses. No one with any knowledge of public affairs would hold the idea that this promise could guarantee for a moment that the

**ROYAL BRITISH NURSES ASSOCIATION**—(*cont.*)

nurses would be on the Register, but 9,000 nurses cannot be ignored.

A Member: I agree with the nurses that, in deciding to amalgamate, one goal was to get State Registration.

Mr. Berkeley: Would it be possible to meet, say, in a week? I think it a thousand pities that Mr. Paterson's reply should be postponed.

Mrs. Latter: We do not wish to stand in the way of what is to be of benefit to both bodies. We will give way to Mr. Paterson if he wishes to go forward with a motion.

Sir James Crichton Browne: I think it is a great pity that we should miss the amalgamation through any delay. Would Mrs. Latter be willing to withdraw her proposal?

Mrs. Latter: I am quite willing to move that my resolution be negatived if Mr. Paterson will go forward with his motion to send the reply drafted to the Privy Council.

Mr. Paterson, on a point of order, asked whether it was in order at the same meeting to negative a resolution already declared to be carried at that meeting.

The Chairman said that it was quite competent for Mrs. Latter to withdraw her amendment by leave of the meeting.

It was then put to the meeting that Mrs. Latter's resolution be negatived. Result, ten for its withdrawal, four against.

Mr. Paterson moved that the letter he had already read be adopted as the reply of the Council to the Privy Council, and this letter was read for the second time.

A Member: Is it agreed that Clause D be altered as suggested by the Privy Council?

Mr. Paterson: No. Clause D stands as printed.

Sir James Crichton Browne: The word "Official" remains, then?

Mr. Paterson: Yes.

Sir James Crichton Browne: Clause D therefore stands as it is, and Mr. Paterson's draft is adopted as a reply to Paragraph 8. But the letter is not in the form of a resolution. I think it ought to be prefixed by a paragraph saying that you accept the other amendments of the Privy Council, and then go on with the letter which may be accepted by the Council as expressing their views. I thought you would accept the amendment in Paragraph D. What more do you want than a Register?

A Member: We want to consider all this first; may we really not be permitted to talk matters over among ourselves? You are all very clever people, and see things quickly and clearly, but we are quite ordinary, and we feel that we need time to think it all over. Will you not allow us to do so? Could not some resolution be proposed that would put this into practice?

Mrs. Latter: I think this is very reasonable.

Mr. Berkeley: We have now a resolution that Mr. Paterson be requested to forward the letter to the Privy Council. Will not someone second this? It was seconded by Miss Chawner and carried *nem. con.*

